# DAX1 promotes cervical cancer cell growth and tumorigenicity through activation of Wnt/β-catenin pathway via GSK3β

**DOI:** 10.1038/s41419-018-0359-6

**Published:** 2018-03-01

**Authors:** Xiao-Fang Liu, Xue-Yuan Li, Peng-Sheng Zheng, Wen-Ting Yang

**Affiliations:** 1 Department of Reproductive Medicine, The First Affiliated Hospital of Xi’an Jiaotong University, 710061 Xi’an, Shaanxi China; 2 Key Laboratory of Environment and Genes Related to Diseases, Ministry of Education of the People’s Republic of China, 710061 Xi’an, Shaanxi China

## Abstract

DAX1 is well known for its fundamental role in several types of cancer, while its biological role in cervical cancer remains largely unexplored. The expression of DAX1 in cervical carcinoma tissue was examined using immunohistochemistry and western blot. The effects of DAX1 silencing on the cell growth, tumor formation, and CSC (cancer stem cell) characteristics were also investigated. DAX1 expressed a gradual increase from normal cervix to high-grade squamous intraepithelial lesions, and consequently to cervical cancer. Silence of DAX1 significantly inhibited the cell growth, tumorigenicity, and tumorsphere formation. Furthermore, the TOP/FOP-Flash reporter assay revealed that Wnt/β-catenin pathway was significantly inactivated in DAX1-silenced cervical cancer cells with the downregulation of Wnt/β-catenin targeting genes, including cyclinD1 and c-myc. Moreover, dual-luciferase reporter and chromatin immunoprecipitation (ChIP) assay confirmed that DAX1 transcriptionally repressed glycogen synthase kinase 3β (GSK3β), an inhibitor of the Wnt/β-catenin pathway, by physically interacting with −666~−444 motif on the GSK3β promoter. Additionally, the blockage of GSK3β by CHIR-99021 resulted in a significant increase of CSC characteristics induced by the silence of DAX1. Our data demonstrated that DAX1 is overexpressed in cervical cancer, and that it promotes cell growth and tumorigenicity through activating Wnt/β-catenin pathway mediated by GSK3β.

## Introduction

Cervical cancer is the fourth most common tumor type and the fourth leading cause of cancer death among women worldwide. An alarming increase in the incidence of cervical cancer has been observed in recent years. Also, nearly 90% of cervical cancer deaths occur in the developing countries^1^. Although the development of cervical cancer is intimately associated with the infection of high-risk human papillomaviruses (HPV), progression from HPV-positive premalignant lesion to invasive carcinoma happens rarely^2^. That is to say, not all patients infected with HPV will develop cervical cancer, or different molecular abnormalities essential for cervical cancer development, like the inactivation of tumor suppressor genes (*TP53*, *CDK2A*, and *PTEN*) or the activation of oncogenes (*RAS* and Wnt pathway), whose underlying mechanisms in cervical cancer have not been clearly illustrated.

DAX1 (also known as nuclear receptor subfamily 0, group B, member 1, Nr0b1) is an unusual member of orphan nuclear receptor, as it contains a conserved LBD, but lacks the canonical zinc-finger-containing DBD. Its N-terminus contains three repeated LXXLL motifs, which mediate the subcellular distribution and nuclear localization of DAX1^3,4^. DAX1 functions primarily as a transcriptional repressor that suppresses the transcriptional activities of hormone NRs (estrogen receptor, ERs, progesterone receptor, PR, and androgen receptor, AR) and many orphan NRs (NR5A1, NR5A2, NR4A1, NR0B2, NR3B3, and NR2A1) through a unique mechanism of protein−protein interaction between DAX1 and DNA-bound NRs^5–8^. Furthermore, DAX1 has the ability to bind to the AF-2 domain of the NRs via N-terminal LXXLL motifs, thereafter directly occupying the coactivator-binding surface and subsequently recruiting co-repressors to the promoters of target genes. Other mechanisms of DAX1-mediated repression include interference with the functional dimerization of NRs, preventing the nuclear translocation of ligand-activated NRs, as well as binding to hairpin elements in the promoter of target genes.

The expression of DAX1 in Ewing’s sarcoma^9^, breast cancer^10^, ovary cancer^11^, endometrial cancer^12^, lung cancer^13,14^, and prostate cancer^15^ has been described, though its expression pattern in cancer progression has shown discrepancy among different types of cancers. Higher expression levels of DAX1 have been found to be correlated with higher rates of lymph node metastasis in lung adenocarcinoma. Moreover, a knockdown of DAX1 can significantly inhibit the invasion capability of lung cancer cells^13^. DAX1 is induced by the oncoprotein chimerical transcription factors (EWS/FLI1); it is highly expressed in Ewing’s tumors and it plays an important role in cell-cycle progression^9^. Also, the tumor-promoting function of DAX1 appears to be context dependent. DAX1 depletion can induce cancer cell migration and potential metastasis in hepatocellular carcinoma where the expression level of DAX1 is downregulated^16^. Nevertheless, the exact function of DAX1 in cervical cancer development is still unclear and needs to be further investigated.

The following section investigates the expression of DAX1 in normal cervix and cervical lesions. It also explores its role in the cervical carcinogenesis by silencing the DAX1 expression in cervical cancer cell lines. Furthermore, this study investigates the mechanical route through which DAX1 causes cervical cancer.

## Results

### Upregulation of DAX1 protein was found in cervical cancer

**Fig. 1:**
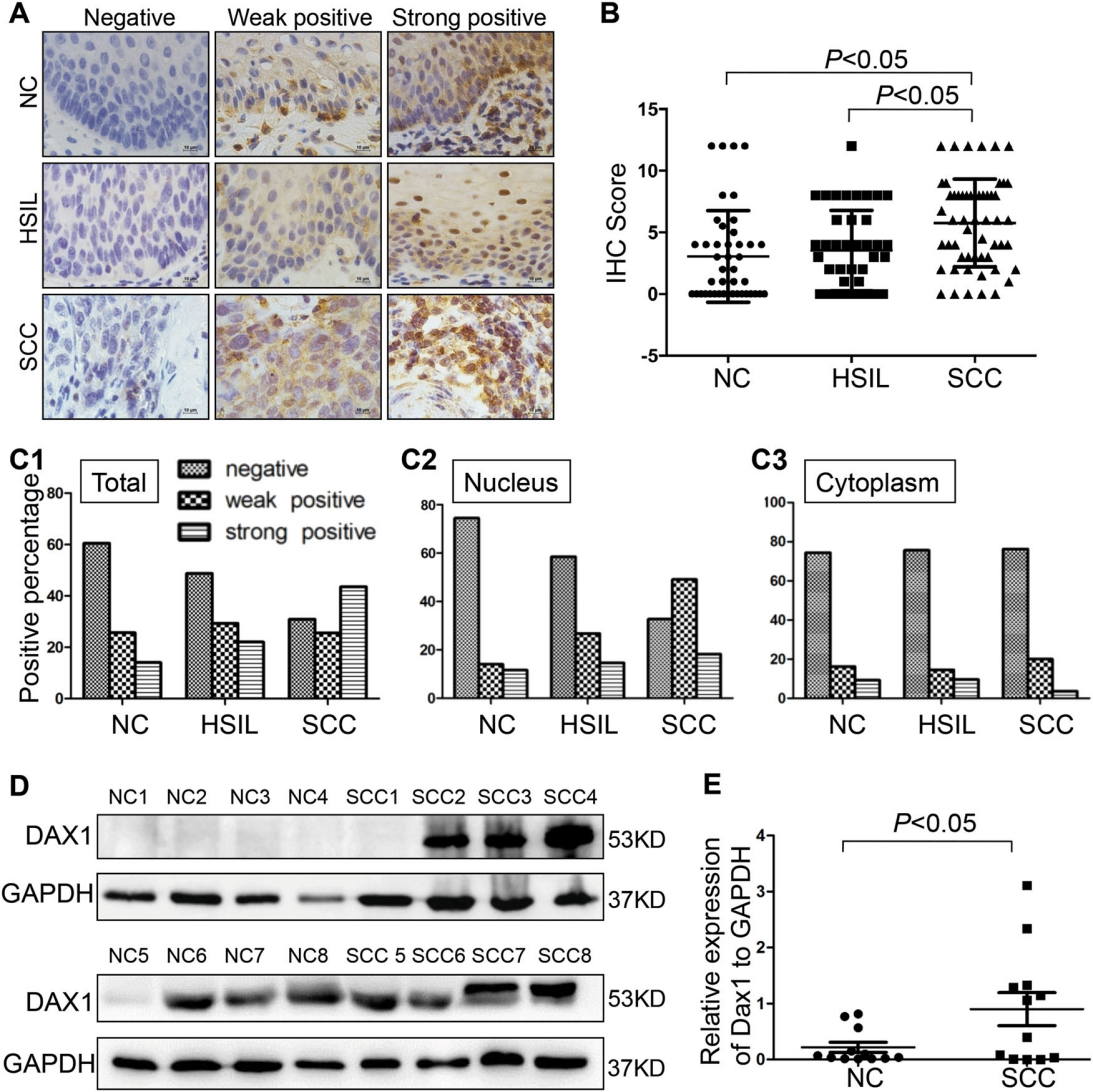
DAX1 expression is shown in normal cervix and different cervical lesions . **a** Immunohistochemistry showing DAX1 expression in 43 NC, 41 HSIL, and 55 SCC. **b** The IHC scores of DAX1 staining in NC, HSIL, and SCC were performed. Data were statistically analyzed with the multiple-comparison test of one-way ANOVA and values are shown as mean ± SD. **c** DAX1 staining is classified into three categories (weak-positive, strong-positive, and negative), and the percentage of each group is shown in total (**c1**), nucleus (**c2**), and cytoplasm (**c3**). **d** Representative western blots of DAX1 protein expression were detected in NC and SCC. **e** Quantitative analysis of DAX1 expression in normal cervix and squamous cervical carcinoma; GAPDH was used as an internal control; Student’s *t*test was carried out. *P < 0.05, **P < 0.01

Using a validated antibody for DAX1, the expression pattern of DAX1 in 43 normal cervical (NC), 41 high-grade squamous intraepithelial lesions (HSIL), and 55 squamous cervical cancer (SCC) stained tissues revealed that DAX1 was located in the nucleus and cytoplasm (Fig. 1a). The analysis of the IHC score showed that DAX1 staining was 3.06 ± 3.72 in NC, 3.54 ± 3.26 in HSIL, and 5.76 ± 3.56 in SCC (*P* < 0.05, Fig. 1b). Also, the total positive (strong-positive and weak-positive) percentage of DAX1 gradually increased from 39.53% (17/43) in NC to 53.66% (22/41) in HSIL, and then to 69.10% (38/55) in SCC (*P* < 0.05, Fig. 1c1). Furthermore, the expression of DAX1 in the nucleus also gradually increased from NC to HSIL and then to SCC (Fig. 1c2). However, the cytoplasm staining of DAX1 was insignificantly different in other level lesions of the cervix (Fig. 1c3).

Meanwhile, the expression of DAX1 protein was detected by western blot in fresh cervical tissues from surgical patients. Briefly, the average relative expression of DAX1 in eight CC was four times higher compared to eight NC (0.94 ± 0.17 vs. 0.21 ± 0.08, *P* < 0.05, Fig. 1d, e). To conclude, the above results indicated that DAX1 was highly expressed in cervical cancer and that it may promote the development and progression of cervical carcinogenesis.

### Silenced DAX1 in cervical cancer inhibits cell growth in vitro and tumorigenicity in vivo

**Fig. 2:**
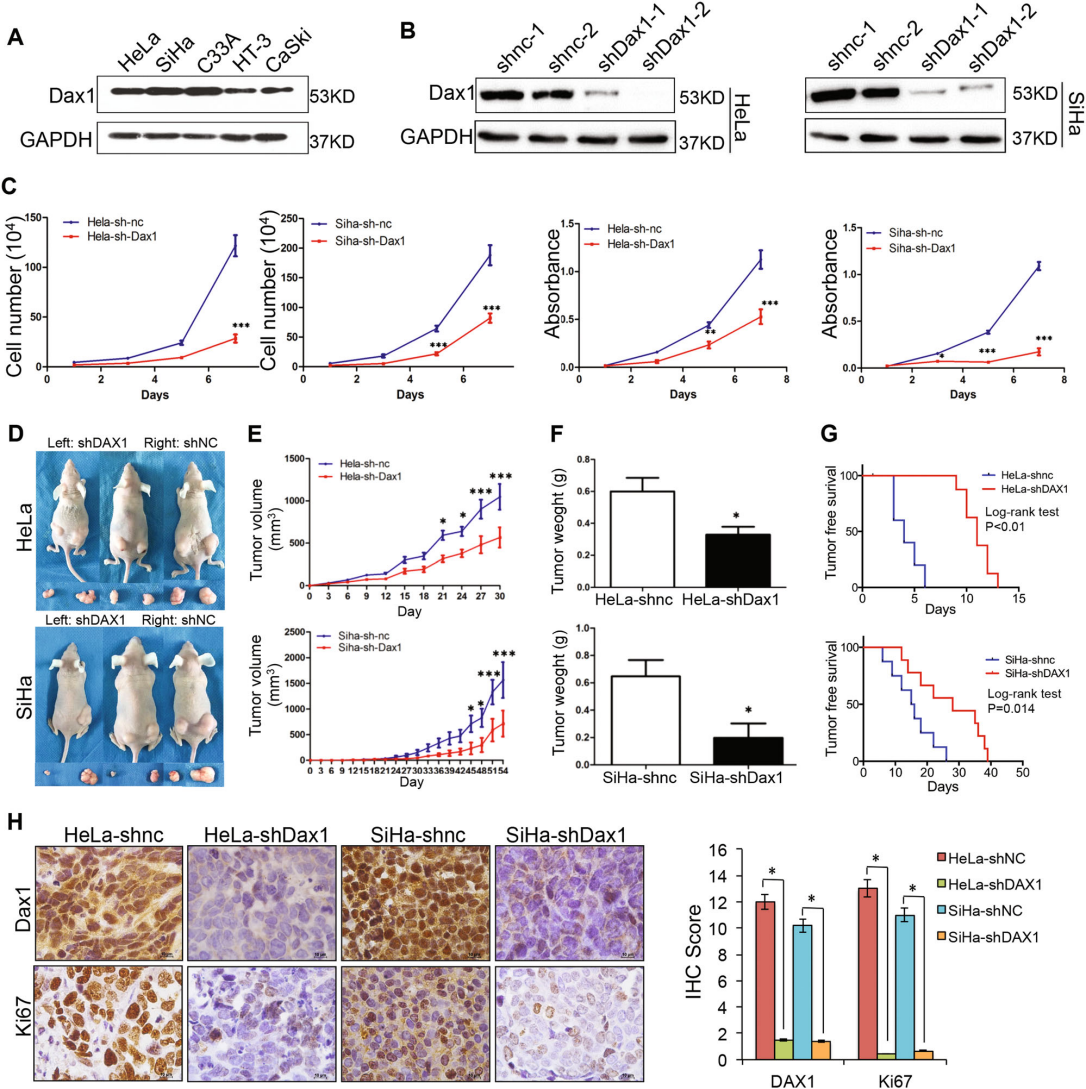
Depletion of DAX1 suppresses the cell growth and tumorigenicity . **a** The expression levels of DAX1 in five cervical cancer cell lines HeLa, SiHa, C33A, HT-3, and CaSki. **b** DAX1-silenced HeLa and SiHa cells were detected by western blot. **c** Cell growth and MTT assays were determined in DAX1-silenced HeLa and SiHa cells. **d**–**g** The tumor growth curve, tumor weight, and tumor-free survival are shown in DAX1-silenced cells and control cells, respectively. **h** The expressions of DAX1 and Ki67 in tumor tissues were in DAX1-silenced cells and control cells. Data were statistically analyzed with Student’s *t*test and values are shown as mean ± SD. *P < 0.05, **P < 0.01, ***P < 0.001

First, high expression of DAX1 was observed in all five cervical cancer cell lines by western blot (Fig. 2a). Furthermore, stably silenced expression of DAX1 in HeLa and SiHa cells was obtained using two different shRNA sequences targeting DAX1 (shDAX1-1 and shDAX1-2, Fig. 2b). Accordingly, the cell growth and cell vitality were significantly inhibited in both DAX1-silenced HeLa and SiHa cells compared to control groups (Fig. 2c, *P* < 0.05). Consequently, 10^5^ cells were implanted in nude mice; and the tumor growth, as well as animal weight, was monitored twice a week until the end of the experiment (Fig. 2d). The results showed that HeLa-shDAX1 and SiHa-shDAX1 xenografts were smaller and lighter compared to the control group (Fig. 2e, f, *P* < 0.05). Additionally, the survival of free tumor in DAX1-silenced cells was significantly longer compared to controls (Fig. 2g, *P* < 0.05). Furthermore, the expression of Ki67, a well-known cell proliferation marker, was weaker in the xenograft tissues formed by DAX1-silenced HeLa and SiHa cells than in those formed by control cells, respectively (Fig. 2h, *P* < 0.05). To sum up, all these results indicated that the silence of DAX1 inhibited the cell growth in vitro and tumor formation of cervical cancer cells in vivo.

To determine whether the tumor promotion of DAX1 was involved in the cell proliferation, cell-cycle distribution was analyzed using FACS, as shown in SFigure 1A and 1B. The percentage of HeLa-shDAX1 cells in G0/G1 phase increased significantly to 62.18%, together with a decrease in the percentage of M-phase cells to 11.46% (ratio of G0/G1 phase to M phase: 62.18%/11.46%, 5.43, *P* < 0.05). A similar effect was observed in SiHa-shDAX1 cells. Furthermore, the transcript levels of cell-cycle-promoting factors, such as cyclinD1, cyclinB, CDK4, CDK2, and CDC2, were all inhibited in DAX1-silenced cervical cancer cells. Conversely, p27, p21, and p16 that served as the inhibitors in cell proliferation showed a higher level in DAX1-silencing cells compared to controls (SFigure 1C). All these results suggested that DAX1 promoted the cell growth and tumor formation of cervical carcinoma possibly by enhancing the proliferation of cervical cancer cells.

### Silenced DAX1 in cervical cancer cells displays decreased characteristics of CSC

**Fig. 3:**
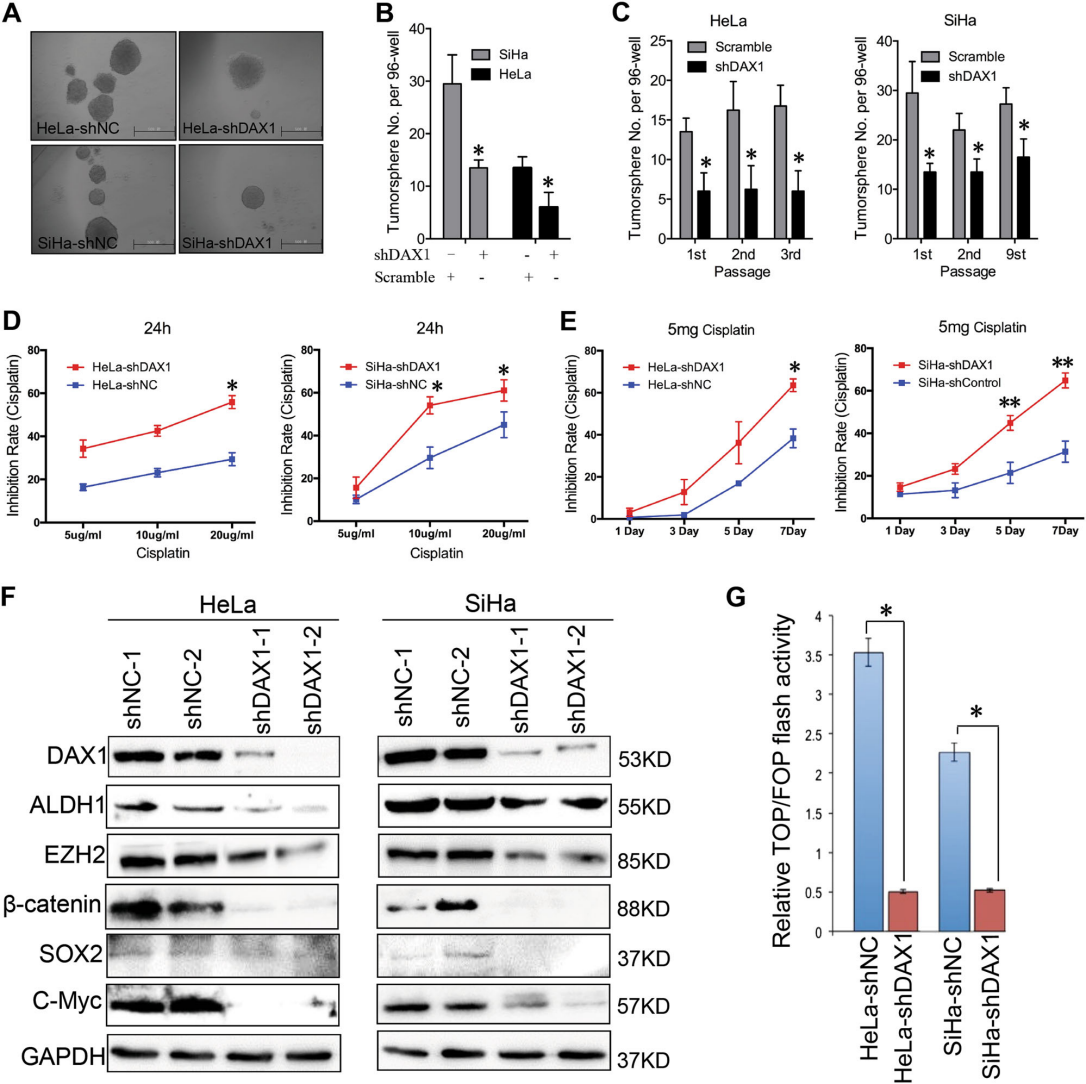
Silence of DAX1 inhibited the CSC characteristics of cervical cancer . **a** Representative photos of tumorspheres formed by DAX1-silenced cervical cancer cells and control cells are shown. **b**, **c** The number of tumorspheres/96 cells was counted from three consecutive passages. **d** Cell viability was measured using an MTT assay after treatment with different concentrations of cisplatin for 24 h. **e** Cell viability was measured using an MTT assay after treatment with a constant dose of cisplatin for 1, 3, 5, and 7 days. **f** Western blot analysis of the protein levels of the stem-cell-associated proteins. **g** DAX1-silenced HeLa and SiHa cells were transfected with the TOP/FOP-Flash reporter plasmid, and the reporter activities were detected 48 h after transfection by a luciferase assay. Data represent mean ± SD of triplicate experiments and were statistically analyzed with Student’s *t*test. *P < 0.05, **P < 0.01

Generally, tumor initiation and growth are driven by a small population of cancer stem cells (CSC). Self-renewal is a critical characteristic of stem cells and CSCs. To assess self-renewal in vitro, DAX1 silencing and control cells were cultured in serum-free medium under conditions optimal for growing tumorspheres (Fig. 3a). As shown in Fig. 3b, when plated at a density of one cell/well in 96-well plates with the limiting dilution assay, the DAX1-silenced HeLa and SiHa cells generated tumorspheres with efficiencies of 12.8 and 5.6%, whereas the control cells generated tumorspheres with the efficiencies of 29.8 and 13.2% in the first passage, respectively (*P* < 0.05). Upon three consecutive passages in culture, the tumorsphere-forming efficiency of DAX1-silenced cells gradually increased (Fig. 3c). These data indicated that the DAX1 inhibited the self-renewal capacity in cervical cancer.

The resistance of CSCs to current chemotherapeutics is thought to be responsible for cancer recurrence and metastasis. Cell viability was determined by the MTT assay after exposure to different concentrations of cisplatin for 24 h. DAX1-silenced cells were significantly less resistant to cisplatin than control cells (Fig. 3d). Then, cells were exposed to a constant concentration of cisplatin for 1, 3, 5, or 7 days (Fig. 3e). Also, DAX1-silenced cells were much less resistant to treatment with cisplatin. These results suggested that DAX1 might promote the resistance ability to chemotherapy.

Stem-cell-related transcription factors are important to maintain self-renewal in embryonic stem cells. DAX1-silenced cells inhibited the expression of ALDH1, EZH2, SOX2, and C-MYC (Fig. 3f). Surprisingly, Wnt/β-catenin signaling, which has a crucial role in cervical carcinogenesis and stem-cell-related pathways^17^, was also inhibited with decreased activation of the TOP-Flash reporter in HeLa and SiHa cells by approximately sevenfold and fourfold, respectively, compared to control groups (Fig. 3g, *P* < 0.05). These data indicated that silence of DAX1 in cervical cancer cells displayed decreased CSC characteristics.

### Silence of DAX1 inhibits Wnt/β-catenin signaling pathway

**Fig. 4:**
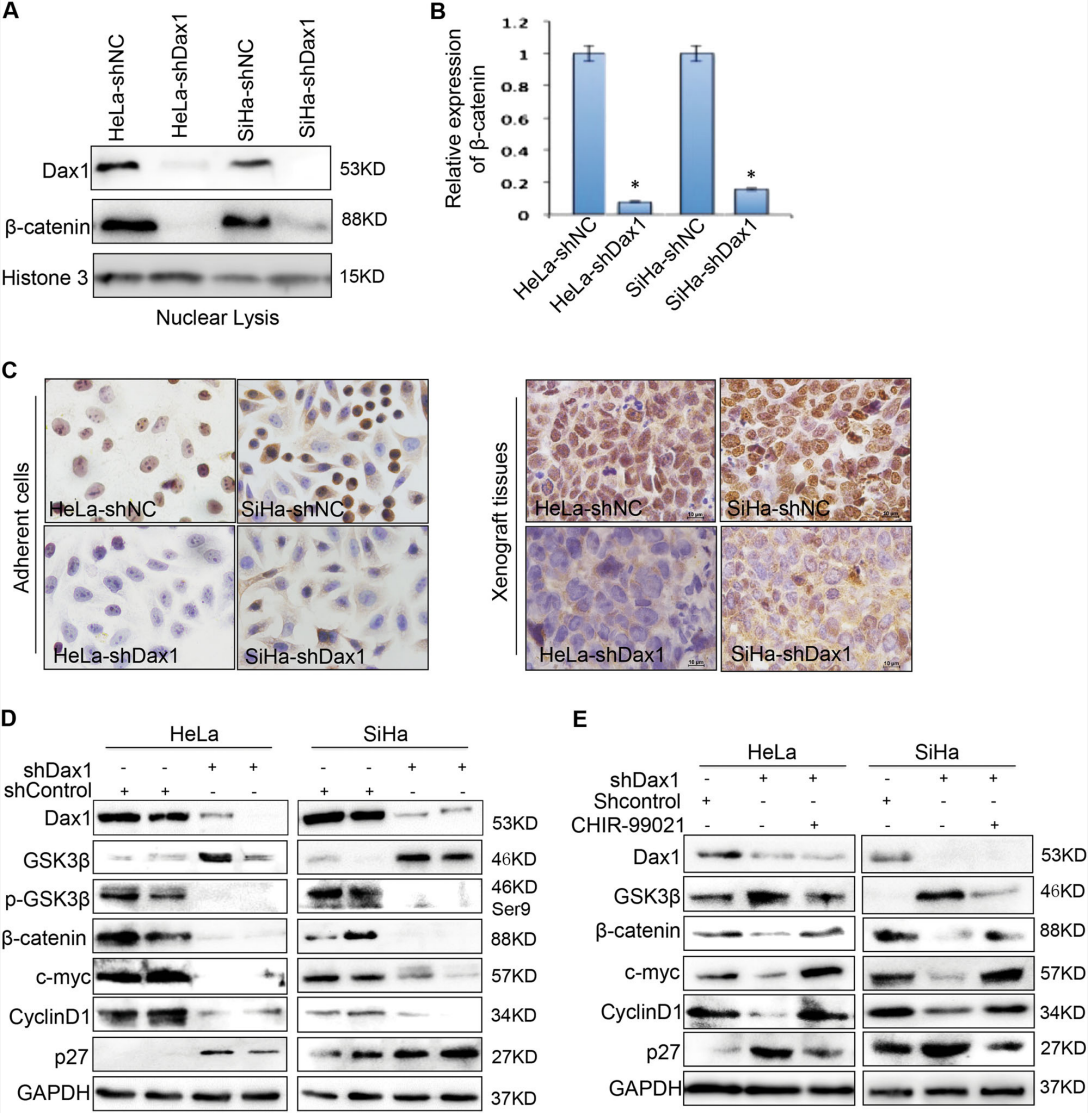
Depletion of DAX1 inactivated the Wnt/β-catenin pathway . **a**, **b** The expression of β-catenin in the nucleus in DAX1-depleted HeLa and SiHa cells was determined by western blot and the quantitative analysis was shown. **c** Immunocytochemistry for β-catenin was performed in DAX1-silenced HeLa and SiHa cells. **d** The expression of β-catenin, GSK3β, c-myc, and cyclinD1 was measured by western blot. **e** GSK3β, β-catenin, c-myc, cyclinD1, and p27 expression were shown after the treatment of GSK3β inhibitor CHIR-99021 in DAX1-knockout HeLa cells and SiHa cells. Data were statistically analyzed with Student’s *t*test and values are shown as mean ± SD. *P < 0.05

In order to confirm that silencing DAX1 inhibited Wnt/β-catenin pathway, by using western blot and IHC, we found that the downexpression of β-catenin and nuclear accumulation (Fig. 4a–c) were accompanied by the downregulation of c-myc and cyclinD1 and upregulation of p27 (Fig. 4d). These results indicated that DAX1 regulates Wnt/β-catenin signaling in cervical cancer cells.

We also investigated whether the β-catenin degradation complex was involved in the cell growth promotion via DAX1/Wnt/β-catenin pathway. GSK3β, a highly effective kinase on a substrate (here referred to as β-catenin), was significantly improved, and the p-GSK3β ^(Ser9)^ in DAX1-knockdown cervical cancer cells eventually led to the degradation of β-catenin in SiHa and HeLa cells (Fig. 4d). Furthermore, CHIR-99021, a pharmacological inhibitor of GSK3β, was used to abolish kinase activity. As expected, CHIR-99021 treatment inhibited the degradation of β-catenin through inhibiting the activation of GSK3β (Fig. 4e). We also sought to determine whether these target genes would change in response to this chemotherapeutic drug. Briefly, Fig. 4e shows that the expression of c-myc and cyclinD1 was increased, while the cell-cycle inhibitor p27 was reduced. This suggested that DAX1 functions as the tumor promoter in cervical cancer, and that it is involved in the activation of Wnt/β-catenin pathway through GSK3β.

### DAX1 inhibits the transcription level of GSK3β by binding to the specific region of GSK3β promoter

**Fig. 5:**
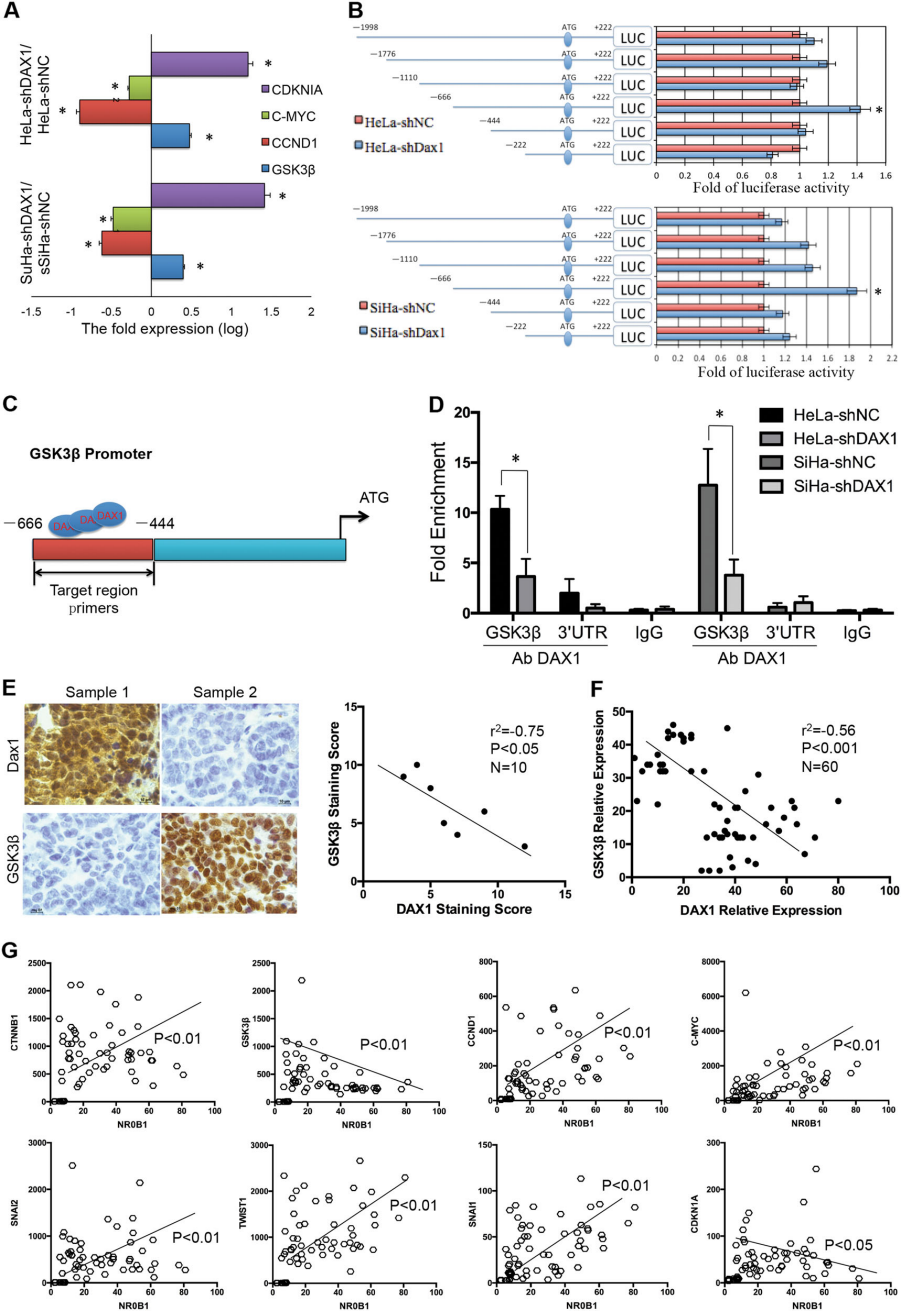
DAX1 activated the Wnt/β-catenin pathway through directly transcriptionally repressing the expression of GSK3β in cervical cancer cells . **a** The transcript levels of GSK3β, CCND1, C-MYC, and CDKNI1A in DAX1-silenced HeLa and SiHa cells were determined by real-time PCR. **b** The GSK3β promoter structure was constructed, and luciferase activity relative to Renilla control was measured in DAX1-silencing HeLa and SiHa cells. **c**, **d** The qChIP assay is shown in the DAX1-knockout HeLa and SiHa cells immunoprecipitation by GSK3β antibody and IgG antibody (as the negative control). **e**, **f** The expression of DAX1 and GSK3β was detected by IHC and real-time PCR; also, the correlation was analyzed. **g** The analysis of correlation between DAX1 and CTNNB1, GSK3β, CCND1, C-MYC, and other target genes of Wnt pathway. Data were obtained from GEO database and correlation analysis used Spearman rank correlation analysis. The two independent samples show mean ± SD and *t*test was used to compare the means of samples. *P < 0.05

To determine whether DAX1 activated Wnt/β-catenin pathway by directly regulating the expression of GSK3β in cervical cancer cells, the silence of DAX1 in HeLa and SiHa cells consistently enhanced the transcriptional level of GSK3β and the target genes (Fig. 5a). These studies revealed that DAX1 actively represses both RNA and protein levels of GSK3β, prompting us to further explore the role of DAX1 in the transcriptional inhibition of GSK3β in cervical cancer cells.

In order to confirm this hypothesis, we performed dual-luciferase reporter system to explore if DAX1 represses the promoter activity of GSK3β. The GSK3β promoter–luciferase reporters were constructed, containing the region from −1998 to +222 upstream of the GSK3β gene ATG. The results showed that the luciferase activity of −666~−444 in both DAX1-knockdown HeLa and SiHa cells was more than two times higher compared to the signals detected in the control cells (Fig. 5b). Next, experiments carried out on 293T cells do not express DAX1, cotransfecting a DAX1 expression vector and the GSK3β reporter vectors, and detected higher levels of repression of GSK3β (SFigure 2A and B). Furthermore, we used the quantitative ChIP (qChIP) assay to identify if DAX1 protein binds to the specific region of GSK3β promoter in vivo. A pair of primers was designed to amplify the GSK3β promoter region from −666 to −444 (Fig. 5c). The immunoprecipitation by DAX1 antibody indicated that enrichment of DAX1 binds to the promoter region of GSK3β (Fig. 5d). In clinical specimens, the expression of DAX1 showed negative correlation with GSK3β both in the protein level and in the transcript level (Fig. 5e, f, *P* < 0.05). These results supported the premise that DAX1 transcriptionally inhibits the expression of GSK3β by directly binding to the promoter of GSK3β in cervical cancer cells.

These observations prompted us to investigate the relevance of DAX1 expression to the Wnt/β-catenin pathway target genes in human cervical cancer tissues. As shown in Fig. 5g, by analyzing the database of GEO (GDS2416, GDS3233, and GDS3292), the expression of DAX1 was also inversely correlated with GSK3β and positively correlated with the expression of CTNNB1 and C-MYC in human cervical tissues (Fig. 5g, *P* < 0.05). Additionally, DAX1 was also positively correlated with other Wnt/β-catenin pathway target genes, including SNAI2, TWIST1, and SNAI1 was negatively correlated with the cell-cycle inhibitor CDKN1A (Fig. 5g).

### Activation of Wnt/β-catenin pathway reversed the inhibition of cell growth and tumorigenicity mediated by DAX1 silencing

**Fig. 6:**
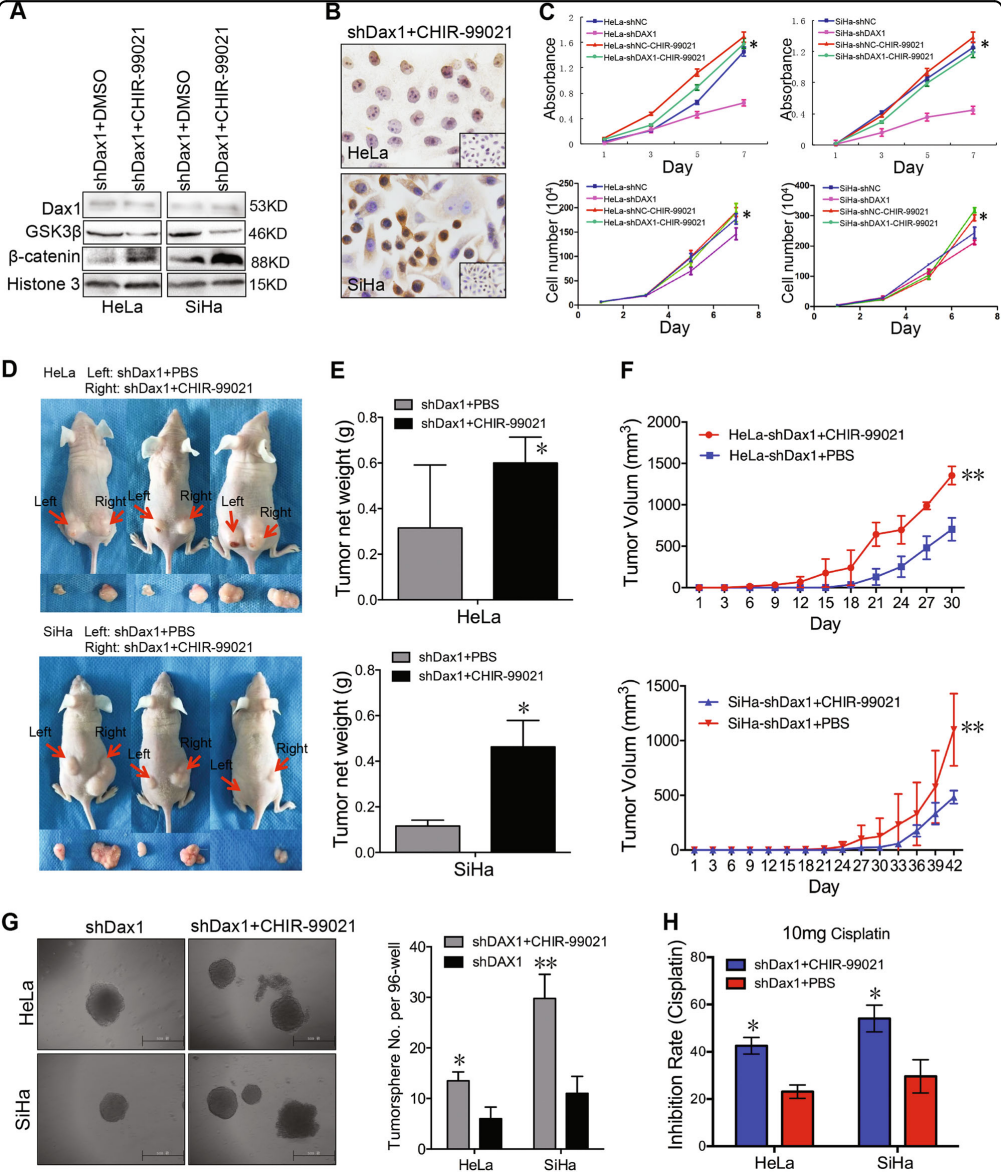
Activation of Wnt/β-catenin pathway reversed the inhibition of cell growth and tumorigenicity mediated by DAX1 silencing . **a**, **b** The expression of β-catenin in the nucleus in DAX1-depleted HeLa and SiHa cells treated with CHIR-99021 was determined by western blot and IHC. **c** Cell growth and viability were measured in DAX1-silenced HeLa and SiHa cells treated by GSK3β inhibitor CHIR-99021. **d**–**f** The mice transplanted with shDAX1 +  PBS of HeLa and SiHa cells group on the left back and shDAX1 + CHIR99021 of HeLa and SiHa cells group on the right back in the same mice by three independent repetitions .(**d**) The tumor growth curve and tumor weight were monitored. **g** Representative photos of tumorspheres and the number of tumorspheres/96 cells were shown. **h** Cell viability was measured using an MTT assay after treatment with CHIR-99021 for 24 h. The data represent mean ± SD from three independent experiments and were statistically analyzed with Student’s *t*test, *P < 0 .05, **P < 0.01

Using CHIR-99021 to abolish the kinase activity of GSK3β in DAX1-silenced cells, Wnt/β-catenin was activated with the increased nuclear accumulation of β-catenin detected by western blot and IHC (Fig. 6a, b). The inhibition of cell vitality and growth mediated by DAX1 silencing was restored in both SiHa and HeLa cells as expected (Fig. 6c, *P* < 0.05). Then, the tumor-formation ability formed by the DAX1-silenced cells treated with CHIR-99021 was increased to the levels of parent cells (Fig. 6d–f, *P* < 0.05) to assess the self-renewal ability affected by CHIR-99021 in DAX1-silenced cervical cancer cells. As shown in Fig. 6g, CHIR-99021 increased the generation of tumorspheres with an efficiency of 13.2 and 30.2%, whereas the DAX1-silenced cells generated tumorspheres with an efficiency of 5.8 and 12.4% in SiHa and HeLa cells, respectively (*P* < 0.05). Also, the activation of Wnt/β-catenin by CHIR-99021 reduced the sensitivity to chemotherapy (Fig. 6h, *P* < 0.05). These results suggested that DAX1 promotes the tumorigenicity and “stemness” through Wnt/β-catenin pathway via GSK3β.

**Fig. 7:**
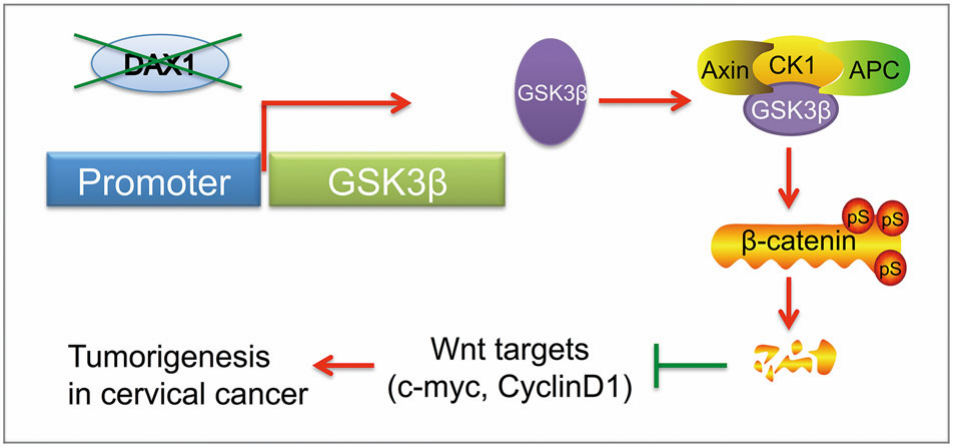
The model of DAX1 functions in cervical cancer . DAX1 transcriptionally repressed the expression of GSK3β to allow β-catenin to escape degradation, which consequently elevated levels of downstream oncogenic genes in cervical cancer, resulting in the promotion of cell growth, tumorigenicity, and CSC characteristics

Taken together, our findings support the notion that overexpression of DAX1 in cervical cancer allows for aberrant binding to the GSK3β promoter, enhancing transcriptional inhibition of GSK3β, which then inhibits the degradation of β-catenin. This allows for the reactivation of Wnt/β-catenin target genes such as c-myc and cyclinD1, which serve to drive cell growth, tumor formation, and CSC characteristics in cervical cancer (Fig. 7).

## Discussion

Thus far, the role of DAX1 in cancer progression has been described in Ewing’s sarcoma and in breast, ovary, endometrium, hepatocellular, lung, and prostate cancers. The expression pattern in the above-reported cancer progressions has shown discrepancy among different types of cancers. A recent study has suggested that DAX1 could inhibit hepatocellular carcinoma proliferation by inhibiting β-catenin transcriptional activity^16^. Also, DAX1 has shown to be expressed in the benign form of breast carcinoma (in situ); and more significantly expressed in invasive breast cancer^18–21^. Therefore, the role and molecular mechanism of DAX1 in the progression of cancer appear to be diverse and complicated, requiring further investigation.

In the present study, our results revealed an important role for DAX1 in cervical cancer progression. We have confirmed that DAX1 was highly expressed in SCC than that in NC. So, we boldly speculated that DAX1 expression levels could be a biomarker for the disease prognosis. However, due to the short follow-up time and better prognosis with postoperative radiotherapy, we did not find the significant difference between DAX1 and the disease prognosis. Loss of DAX1 led to inhibited cervical cancer growth in vitro and in vivo and the characteristic of CSC. By developing a novel context, we demonstrated that ablation of DAX1 potently deactivated Wnt/β-catenin pathway, thus reducing cervical cancer progression. Mechanistically, loss of DAX1 resulted in upregulation of GSK3β by transcriptional inactivation. We further showed that DAX1 could bind to the promoter of GSK3β, thus identifying DAX1 as an oncogenic driver in cervical carcinogenesis.

There is some evidence that activation of Wnt/β-catenin may result from a more general mechanism that follows confusion of nuclear receptors. Orphan nuclear receptor estrogen-related receptor gamma (ERR-gamma) induces GSK3β phosphorylation by upregulating PLK2 responsible for the differentiation of neuroblastoma SH-SY5Y cells. The interplay between NGFI-B and Wnt signaling pathway can disrupt the association between β-catenin and TCF-4, and recruitment of transcriptional co-repressors to the promoters of Wnt targets. Additionally, RORα binds to β-catenin that suppresses Wnt/β-catenin gene transcription, known to be an important pathway in breast cancer cell adhesion and cell growth. Thus, DAX1, as a member of orphan receptor, might influence the Wnt/β-catenin through protein−protein interaction or through recruitment of co-repressors to the promoters of target genes in cervical cancer.

DAX1 could act as a co-repressor for several transcription factors and nuclear receptors, especially hormone NRs and many orphan NRs. The mechanisms of DAX1-mediated repression include a unique mechanism of protein−protein interaction between DAX1 and DNA-bound NRs. They directly occupy the coactivator-binding surface and subsequently recruit co-repressors to the promoters of target genes, by interfering with the functional dimerization of NRs, as well as binding to hairpin elements in the promoters of target genes^4^. In cancers, DAX1 might act as a tumor suppressor by inhibiting β-catenin transcriptional activity. However, our findings suggested that DAX1 promoted the nuclear translocation of β-catenin through repressed activation of GSK3β, APC, and Axin multiprotein complex by transcriptionally inhibiting the expression of GSK3β. In addition, in the GSK3β promoter, we identified that DAX1 binds to the site of −222~−444 by qChIP assay. Further studies are necessary to determine whether co-repressors are involved in the DAX1-mediated repression of GSK3β. Additionally, based on the potential role of Wnt/β-catenin pathway, inhibitors for the clinical tumor therapy, and also Wnt/β-catenin pathway were shown to be significantly activated with the high expression of β-catenin, supported by DAX1, as the nuclear receptor might be the therapeutic target.

To conclude, in the present study, we found that DAX1 expression was upregulated in cervical cancer tissues and cells. Silence of DAX1 inhibited the cell growth and tumorigenicity. Furthermore, we discovered that DAX1 transcriptionally repressed the expression of GSK3β, thus allowing β-catenin to escape degradation, which consequently elevated the levels of downstream oncogenic genes. Therefore, our results identified a novel role for DAX1 in the regulation of cervical cancer development.

## Materials and methods

### Human tissue specimens

A total of 43 NC, 41 HSIL tissues, and 55 SCC samples were collected from the First Affiliated Hospital of Xi’an Jiaotong University from 2009 to 2016. All the procedures followed approved medical ethics practices. None of the patients had received chemotherapy, immunotherapy, or radiotherapy before the specimens were collected. The histological classifications and clinical staging were carried out in accordance with the International Federation of Gynecology and Obstetrics classification system. The institutional review board named as Ethics Committee of Medical School of Xi’an Jiaotong University in Shannxi, China approved the population study, while all the patients provided their informed consent prior to specimen collection.

### Immunohistochemistry and immunocytochemistry

The immunohistochemical staining procedure was performed as previously described^22^. The DAX1 staining (K-17, SC-841, Santa Cruz, CA, USA, dilution 1:200) was classified into three categories: negative, weak-positive, and strong-positive expression, based on the percentage of positive cells and the staining intensity. The percentage of positive cells was divided into five score ranks: <10% (0), 10−25% (1), 25−50% (2), 50−75% (3), and >75% (4). The intensity of staining was divided into four score ranks: no staining (0), light brown (1), brown (2), and dark brown (3). The positivity of DAX1 staining was determined by the following formula: immunoreactivity score (IRS) = intensity score × quantity score. The overall score of ≤3 was defined as negative, >3 but ≤6 as weak-positive, and >6 as strong-positive. Two different pathologists evaluated all the specimens in a blinded manner.

For the immunocytochemistry experiments, cells were cultured on coverslips, fixed with 4% paraformaldehyde for 30 min at room temperature, permeabilized with 0.2% Triton X-100 for 15 min at room temperature, and then incubated with the primary antibodies described above. All antibodies against DAX1 (K-17, SC-841), Ki67 (KI-67, SC-23900), β-catenin (10H8, SC-65480), cyclinD1 (6D328, SC-70899), c-myc (9E10, SC-40), and GSK3β (1V001, SC-71186) were obtained from Santa Cruz Biotechnology (Santa Cruz, CA, USA).

### Cell lines and cell culture

The human cervical cancer cell lines (HeLa, SiHa, C33A, CaSki, and HT-3) were purchased from American Type Culture Collection (ATCC; Manassas, VA). The HeLa, SiHa, and C33A cells were cultured in Dulbecco’s modified Eagle’s medium (DMEM; Sigma-Aldrich, St Louis, MO, USA); CaSki and HT-3 cells were cultured in RPMI-1640 (Sigma-Aldrich) and McCoy’s 5A medium (Sigma-Aldrich), respectively, supplemented with 10% heat-inactivated fetal bovine serum (Hyclone, Thermo Scientific, Waltham, MA, USA). All cell lines were maintained at 37 °C with 5% CO_2_.

### Vector construction and transfection

The small interfering RNA expression vector that expresses DAX1-specific short hairpin RNA (shRNA) with the vector bone of pGPU6/GFP/Neo was purchased from GenePharma Co., Ltd (C02007, GenePharma, Shanghai, China).

Cervical cancer cell lines, SiHa, and HeLa were transfected using Lipofectamine 2000 reagent (Invitrogen, Carlsbad, CA, USA) according to the manufacturer’s instructions. The transfected cells were treated with G418 (Calbiochem, La Jolla, CA, USA) for approximately 3 weeks, and consequently, the drug-resistant colonies were collected, expanded, and identified.

### Cell growth and cell viability assays

A total of 4×10^4^ cells were mixed with 1.5 ml of media and seeded in triplicate in 35-mm culture dishes for 7 days. The numbers of cells were counted after being harvested every 2 days using a hemocytometer under light microscopy. The cell viability assays were performed by applying 3-(4,5-dimethylthiazole-yl)-2,5-diphenyl tetrazolium bromide (MTT; Sigma-Aldrich) dye to cells that were seeded in 96-well plates for 1000 cells each, as described in a standard protocol. Then, the absorbance was measured at 490 nm by BioTek Microplate Instrumentation.

### Tumorsphere culture

Cells were maintained in stem cell media consisting of DMEM/F12 basal media, N2, and B27 supplements (Invitrogen), 20 ng/mL human recombinant epidermal growth factor, and 20 ng/mL basic fibroblastic growth factor (PeproTech Inc., Rocky Hill, NJ). For the tumorsphere formation assay, cells were plated at a density of 200 cells/well in 24-well ultra-low-attachment plates or at a density of one cell/well in 96-well plates and maintained in stem cell medium. Tumorspheres that arose within 2 weeks were recorded. For serial tumorsphere formation assays, the spheres were harvested, disaggregated with 0.25% trypsin/EDTA, filtered through a 40-μm mesh, and replated as described above. For each cell type, triplicate samples were used, and the spheres were counted by two individuals in a blind fashion.

### Flow cytometry analysis

The cell-cycle analysis was performed using FACS (Becton Dickinson, Franklin Lakes, NJ) according to the manufacturer’s protocol. Briefly, the cells were harvested with a concentration of 10^6^ and fixed in 75% ethanol overnight at 4 °C. Thirty minutes before FACS analysis, the cells were treated with RNaseA (Sigma-Aldrich) and then stained with propidium iodide (Sigma-Aldrich). Cell-cycle distribution was analyzed with FACSCalibur flow cytometer and using Flow Jo software.

### Dual-luciferase reporter assay

TOP/FOP-Flash reporter and pTK-RL plasmids (21–170, Millipore, Temecula, CA, USA) were transiently cotransfected into tumor cells (5×10^4^) plated in the 24-well plate dish, while the activity of both firefly and *Renilla* luciferase reporters was determined 48 h post transfection using the Dual Luciferase Assay kit (Promega, Madison, WI, USA), according to the manufacturer’s instructions. The TOP/FOP-Flash reporter activity was presented as the relative ratio of firefly luciferase activity to *Renilla* luciferase activity. All experiments were performed in triplicate.

GSK3β promoter reporter plasmids were constructed (the pGL3 reporter vectors were purchased from Promega, E1751). Plasmids containing firefly luciferase reporters of GSK3β promoter and pTK-RL plasmids were cotransfected into DAX1-silenced SiHa and HeLa cells and the control cells, respectively. After being incubated for 48 h, the cell monolayers were harvested by resuspension in passive lysis buffer. Luciferase signal was detected using luminometer (Promega). The efficiency of transfection was normalized to the paired *Renilla* luciferase activity using the Dual Luciferase Reporter Assay System (Promega). The specific activity was displayed as the fold change of the experimental group versus the control group.

### Quantitative chromatin immunoprecipitation

Quantitative chromatin immunoprecipitation (qChIP) assays were performed according to the manufacturer’s protocol for the EZ-ChIPTM Assay Kit (Cat#17–371; Millipore). Regions of interest were amplified from precipitated samples by real-time polymerase chain reaction (PCR). Each sample was assayed in triplicate, and the amount of precipitated DNA was calculated as a percentage of the input sample. The primers used in quantitative ChIP assays are listed as following:

GSK3β-ChIP F: cagcgctttatagagacgccctc, GSK3β-ChIP R: gagttgaggacggtgaaaccc

GSK3β-3′UTR F: cctcccacaacacgtaggaatt, GSK3β-3′UTR R: ttgctaggaccaaccacatcaa

### Western blotting

Western blot analyses were performed as previously described using 30 μg of lysates from fresh tissues and cells^23^. The primary antibodies were anti-DAX1 (1:1000, Santa Cruz, CA, USA), anti-GAPDH (1:1000, Santa Cruz, CA, USA), anti-β-catenin (1:1000, Santa Cruz, CA, USA), anti-c-myc (1:500, Santa Cruz, CA, USA), anti-cyclinD1 (1:500, Santa Cruz, CA, USA), anti-GSK-3β, anti-p-GSK-3β (1:1000, Santa Cruz, CA, USA), and anti-p27 (1:1000, Santa Cruz, CA, USA). The secondary incubation antibodies used a horseradish peroxidase-conjugated anti-rabbit or anti-mouse IgG (Thermo Fisher Scientific, New York, NY, USA). The signals were then detected by enhanced chemiluminescence reagent (Millipore, Billerica, MA, USA).

### Tumor xenograft experiment

Four- to 6-week-old female BALB/c-nude mice were obtained from Shanghai Slac Laboratory Animal Co. Ltd, China. Briefly, two groups of control and DAX1-silenced SiHa and HeLa cells in the exponential growth phase were harvested for inoculation and consequently implanted by subcutaneous injection (1×10^6^/mice) in the dorsal side of the upper hind limb. The tumor volume (V) was determined by the tumor length (*a*) and width (*b*) as *V* = *ab*^2^/2. All animal studies (including the mice euthanasia procedure) were carried out in compliance with the regulations and guidelines of Xi’an Jiaotong University Institutional Animal Care and conducted according to the AAALAC and the IACUC guidelines.

### Statistical analysis

Statistical analysis was performed with SPSS 18.0 software (SPSS Inc., Chicago, IL, USA). Measurement data were analyzed with mean ± standard deviation; the groups were analyzed using one-way ANOVA analysis, while post hoc test was performed for comparison among groups. Consistent with the measurement data, *t*test was used for normal distribution, while the Wilcoxon rank sum test was used for not normally distributed data; chi-square test was used for count data. *P* value < 0.05 was considered statistically significant.

## Electronic supplementary material

SFigure 1

SFigure 2

STable-primers

Supplementary Figure Legends
