# Supplementary figures and images for: DAX1 promotes cervical cancer cell growth and tumorigenicity through activation of Wnt/β-catenin pathway via GSK3β
Source: Cell Death Dis. 2018 Mar 1;9(3):339. doi: 10.1038/s41419-018-0359-6 (PMC5832878; doi:10.1038/s41419-018-0359-6)

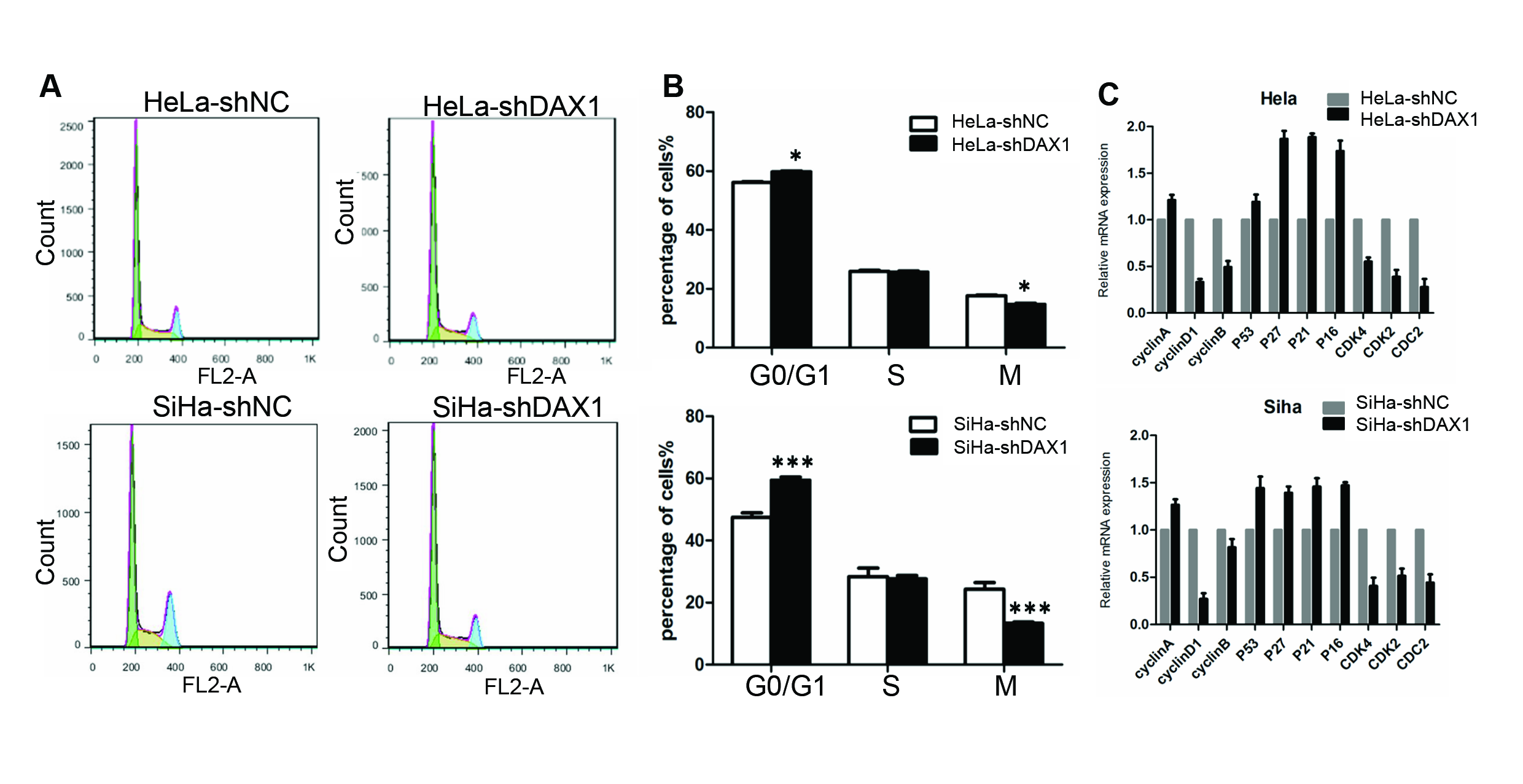

Supplement: Supplementary file 1 — SFigure 1 [file 41419_2018_359_MOESM1_ESM.tif]

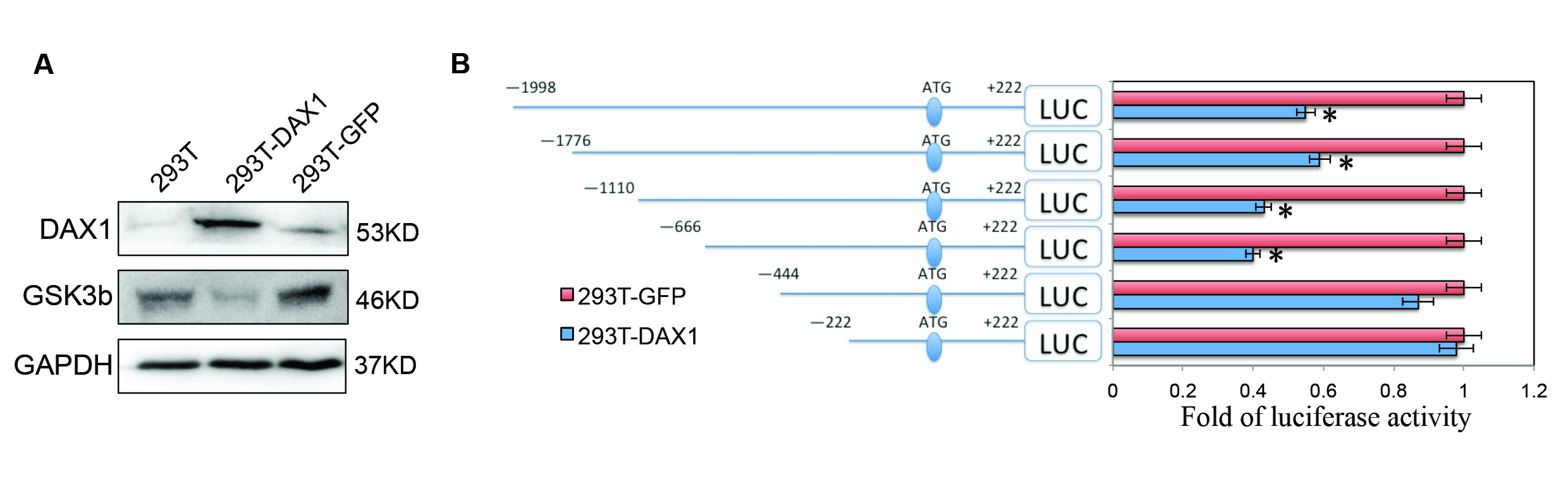

Supplement: Supplementary file 2 — SFigure 2 [file 41419_2018_359_MOESM2_ESM.tif]
