# Supplementary material for: DAX1 promotes cervical cancer cell growth and tumorigenicity through activation of Wnt/β-catenin pathway via GSK3β
Source: Cell Death Dis. 2018 Mar 1;9(3):339. doi: 10.1038/s41419-018-0359-6 (PMC5832878; doi:10.1038/s41419-018-0359-6)
Supplement: Supplementary file 4 — Supplementary Figure Legends [file 41419_2018_359_MOESM4_ESM.docx]

**Supplementary Figure Legends**

**SFigure 1. The distribution of cell cycle in DAX1 silenced cells.** (A and B) The cell cycles were detected by FACS analysis. A quantitative analysis of the cell cycle distribution is shown, respectively, in DAX1-silenced SiHa and HeLa cells. (C) The cell cycle related genes transcripts were detected by Real-time PCR. Values are shown as the mean±SD. * *P*<0.05.

**SFigure 2. The luciferase assay was performed in 293T cells.** (A) The expressions of DAX1 and GSK3β were detected in transient transfected 293T cells. (B) The luciferase assay was performed in 293T cells through co-transfecting the DAX1 overexpression vector and the report vectors. Values are shown as the mean±SD. * *P*<0.05.
